# A Stepped-Wedge Social Media Training Intervention for Community Health Workers In Spanish-Speaking Communities: A Study Protocol for Dime La VerDAD

**DOI:** 10.21203/rs.3.rs-9601998/v1

**Published:** 2026-05-07

**Authors:** Regina Royan, Aashna Sunderrajan, Yajaira Bolanos Flores, Marla L. Clayman, Susan Lopez, Patrick Ten Eyck, Lisa Aponte-Soto, Kang Zhao, De Shauna Jones, Anindita Bandyopadhyay, Vineet M Arora, Marina Del Rios

**Affiliations:** University of Michigan–Ann Arbor; University of Chicago; University of Chicago; Veterans Health Administration; Rush University; University of Iowa; Tanoma Consulting LLC; University of Iowa; University of Iowa; University of Iowa; University of Chicago; University of Chicago

**Keywords:** Social media, Health communication, Community health workers, Promotores de salud, Vaccination, Science communication, Spanish-speaking communities, Public health intervention

## Abstract

**Background:**

Social media has become a central channel for the dissemination of health information, enabling rapid sharing of evidence-based guidance while also facilitating the spread of inaccurate or misleading content. Exposure to such information has been associated with changes in vaccination-related attitudes and decision-making. These effects may not occur uniformly across populations, and there is limited understanding of how health information circulates within Spanish-language social media networks or which communication strategies are most effective in promoting engagement and informed decision-making. This study describes a protocol to evaluate a community-engaged intervention designed to address these gaps.

**Methods:**

This study will use a non-randomized stepped-wedge design to evaluate the implementation and effectiveness of the Dime La VerDAD intervention across community-based cohorts of promotores de salud in Chicago. Promotores will be grouped into geographically defined clusters and will transition from control to intervention at six-month intervals, such that all clusters receive the intervention by study end. The intervention will consist of a structured, bilingual training program focused on identifying inaccurate health claims, evaluating source credibility, and developing accessible, evidence-based social media content, including narrative-based messaging. Outcomes will be assessed using surveys, focus groups, social media analytics, and publicly available epidemiologic data. Primary and secondary outcomes will include changes in knowledge, communication practices, engagement with social media content, and vaccination-related decision-making. Analyses will use mixed-effects models to evaluate changes over time while accounting for clustering and repeated measures.

**Discussion:**

This study will generate evidence on how health information is shared and interpreted within Spanish-language social media networks and evaluate whether a structured, community-engaged communication intervention improves the quality and reach of health messaging. Findings will inform the development of scalable, community-based strategies to support dissemination of reliable health information and promote informed decision-making in diverse populations.

**Trial registration:**

ClinicalTrials.gov NCT06417762. Recruitment has begun and is ongoing at the time of manuscript submission.

## Background

Social media has become a primary channel for the dissemination of health information, enabling the rapid sharing of evidence-based medical guidance across broad audiences. At the same time, these platforms also facilitate the widespread circulation of inaccurate or misleading health information, shaping how individuals access, interpret, and act on health content [1]. During the COVID-19 pandemic, vaccine-related discussions expanded substantially across social media, accelerating both the volume and visibility of health-related messages and contributing to uncertainty about recommended immunizations [2]. As social media continues to play a central role in health communication, understanding how health messages are conveyed and received in these environments is critical for public health.

Exposure to inaccurate or misleading health information on social media has been associated with measurable changes in vaccination-related attitudes and decision-making [3, 4]. Such exposure can shape how individuals interpret risk, assess the credibility of scientific evidence, and determine whether to pursue recommended immunizations. During the COVID-19 pandemic, for example, widespread circulation of conflicting health claims on social media was associated with changes in perceptions of vaccine safety and reduced adherence to public health guidance, particularly in the context of uncertainty and rapidly evolving evidence [5–8]. Social media has similarly emerged as a key source of vaccine-related information for seasonal influenza, where variation in message content and source credibility has been associated with differences in vaccination behavior and uptake [6–9]. These patterns thus underscore the importance of understanding how health information is encountered and interpreted within social media environments.

However, the effects of health information on social media may not occur uniformly across populations. Differences in social networks, access to information, and trusted sources can shape how health information is accessed, interpreted, and shared across communities [10–12]. These considerations are particularly relevant for Spanish-speaking communities, where the pathways through which health information circulates, the sources perceived as credible, and the reception of health information have not been well characterized [13–15]. For example, studies have shown that Spanish-speaking individuals may face challenges accessing trusted health information and often rely more heavily on social media and interpersonal networks for health-related content [16]. Levels of trust in health information sources also vary substantially within Spanish-speaking communities, further shaping how health messages are received and acted upon [17]. Understanding these differences is essential for interpreting how health information circulates within and across social media networks [18, 19].

In this context, understanding which characteristics of health messages influence how information is interpreted and shared on social media is also critical. Prior research suggests that message format plays an important role in shaping engagement and comprehension, with health messages that incorporate personal narratives often perceived as more relatable and easier to understand [20]. In addition to message characteristics, the credibility of the information source influences how messages are received and acted upon. Information delivered by trusted community communicators can shape attitudes and information-sharing behaviors, particularly when messages are conveyed by individuals with established relationships within their communities [21]. For example, collaborative efforts between healthcare professionals and community-based communicators have demonstrated the feasibility of producing culturally and linguistically appropriate health content for Spanish-speaking communities [22–24]. Similarly, in Illinois, promotores de salud (community health workers) have played an important role in disseminating health information within Spanish-speaking communities, including during the COVID-19 pandemic.[25][21] These findings highlight the importance of both message characteristics and source credibility in shaping how health information is understood and shared.

Despite this work, there remains a limited understanding of how health information circulates and is interpreted within Spanish-language social media networks. In particular, few studies have examined how inaccurate or misleading health information is encountered in these settings or how responses grounded in scientific evidence are received by community members. In addition, it remains unclear which communication approaches are most effective in promoting engagement, recall, and informed decision-making in real-world social media environments. Addressing these gaps is essential for developing effective and scalable approaches to communicating evidence-based health information within Spanish-speaking communities.

To address these gaps, we developed Dime La VerDAD (“Tell Me the Truth”: Verify, Debunk, and Disseminate), a structured, community-engaged social media training program designed to support promotores de salud in disseminating reliable, evidence-based information about respiratory virus prevention. The program is grounded in principles of health communication and is designed to build practical skills in identifying inaccurate health claims, developing clear and accessible messages, and engaging effectively with social media audiences.

This study will evaluate the implementation and effectiveness of the Dime La VerDAD program using a stepped-wedge design across community-based cohorts. Specifically, it will characterize how health information is shared and interpreted within Spanish-language social media networks, evaluate whether a structured, evidence-based communication training program improves the quality and reach of health messaging, and assess whether narrative-based approaches differ from standard informational content in influencing engagement and vaccination-related decision-making. We hypothesize that promotores will be perceived as trusted messengers and that narrative-based posts will result in greater engagement and improved decision-making in favor of recommended immunizations.

## Methods

### Study Design

This study will use a non-randomized, stepped-wedge design to evaluate the implementation and effectiveness of the Dime La VerDAD intervention across community-based cohorts of promotores de salud. Promotores will be enrolled in clusters defined by the geographic areas in which they provide services. Three clusters will include promotores serving two geographically adjacent communities, and one cluster will include promotores serving a single, geographically distinct area. Each cluster will include approximately 5–14 promotores, depending on the number of communities represented, with larger clusters corresponding to areas serving multiple adjacent neighborhoods. This cluster structure was informed by pilot data indicating that promotores frequently serve multiple neighboring communities; grouping adjacent areas is intended to reduce contamination across study conditions and minimize spillover effects.

At six-month intervals, each cluster will transition from the control condition to the intervention, such that all clusters will receive the intervention by the end of the study period. During the control phase, promotores will continue their usual social media practices without structured training or guidance from the study team. Upon transition to the intervention phase, participants will complete a structured science communication curriculum and apply these principles by developing and sharing social media content within their networks.

Outcomes will be compared within clusters over time, with each cluster serving as its own control, and between clusters as sequential crossover to the intervention occurs. This design allows for both within-cluster and between-cluster comparisons while accounting for secular trends in social media use and vaccination-related attitudes.

### Study Setting

**Figure 1:**
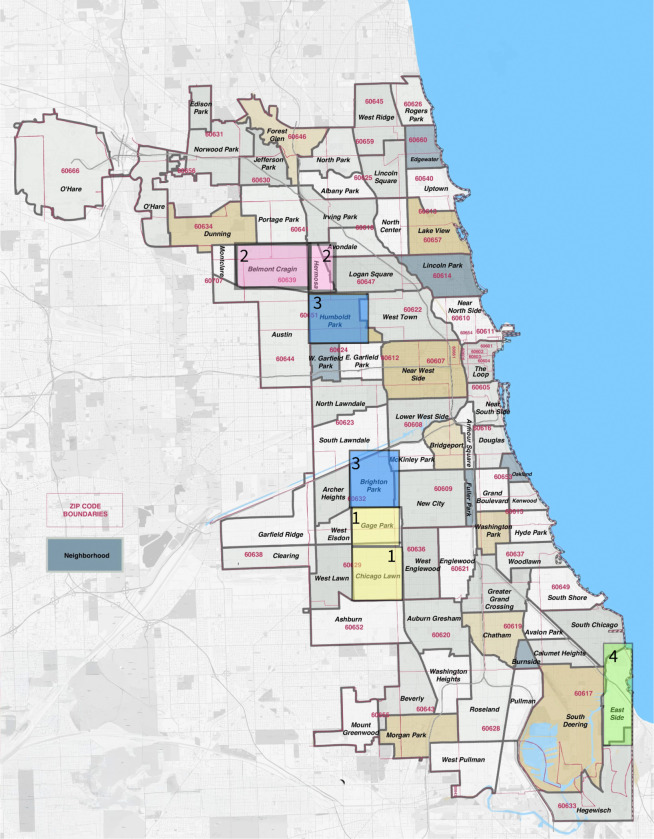
Geographically distinct community areas used for the recruitment of promotores.

This study will be conducted in community settings across selected neighborhoods in Chicago characterized by lower immunization coverage and higher rates of respiratory virus infection [26]. Zip codes and community areas of interest are shown in Fig. 1 and include Gage Park, Chicago Lawn, Hermosa, Belmont Cragin, Humboldt Park, Brighton Park, and Eastside, which were designated under the Protect Chicago Plus program in response to the COVID-19 pandemic [27].

Study activities will take place in person at promotores’ worksites or community locations, as well as virtually via Zoom using bilingual (English and Spanish) instructors.

### Participants

We will recruit promotores de salud from community-based organizations serving the designated study areas. Eligible participants will include promotores who are 18 years of age or older, fluent in English or Spanish, provide services in at least one of the designated community areas, and maintain a personal or work-related social media presence. Promotores will be excluded if they plan to discontinue their work prior to completion of data collection, are unable to communicate in English or Spanish, do not serve the designated community areas, or decline participation in study-related social media activities.

Promotores will be recruited through established partnerships with community-based organizations. Recruitment strategies will include distribution of study materials through organizational networks, presentations at community meetings, and referral through organizational leaders. Promotores may also self-nominate or be referred by community-based partners. Only individuals who directly express interest or provide contact information for follow-up will receive recruitment communications from the study team. Interested individuals will be screened by telephone to confirm eligibility and enrolled into cohort groups based on the communities they serve. Purposive sampling will be used to prioritize bilingual (English/Spanish) or Spanish-speaking promotores serving the designated community areas.

Participation in the study will be voluntary for all participants. Promotores will be provided with an information sheet describing study procedures, potential risks and benefits, confidentiality protections, and data-sharing plans prior to participation in any study activities. Members of the research team will review this information with participants and provide opportunities to ask questions. Consent will be indicated through voluntary participation in study activities, including surveys, focus groups, and social media engagement. Participants may decline specific activities or withdraw from the study at any time without penalty.

Members of promotores’ social media networks will be recruited through posts shared within existing social media platforms. Interested individuals will be directed to a survey link or QR code, where they will receive an electronic information sheet describing the study purpose, voluntary nature of participation, data to be collected, and confidentiality protections. Completion of the survey will indicate implied consent. No consent language will be posted publicly on social media platforms.

Promotores who discontinue participation will be invited to participate in an optional feedback session to identify barriers to engagement. These sessions will be facilitated by study staff not involved in curriculum delivery to encourage open discussion.

Promotores will be compensated at a rate of $30 per hour for structured activities, including surveys, focus groups, and training sessions. During the campaign period, participants will also receive $30 per month for ongoing activities, including social media posting and sharing engagement data. Members of promotores’ social media networks will receive $10 gift cards for each completed survey (baseline, mid-campaign, and post-campaign), with an additional incentive provided for completion of the final follow-up survey.

### Intervention

The Dime La VerDAD intervention will be a structured, community-engaged social media training program designed to support promotores de salud in disseminating reliable, evidence-based information about respiratory virus prevention within their social media networks. The program will be grounded in principles of health communication and will focus on building practical skills in identifying inaccurate health claims, evaluating information sources, and developing clear and accessible health messages.

The curriculum will include training in infographic design, assessment of source credibility, and strategies for communicating effectively across diverse audiences. Participants will be guided in incorporating personal narratives into their social media posts and in responding constructively to disagreement in online environments. As part of the program, promotores will also develop original social media content, including infographics and accompanying captions, and will share these posts within their existing social media networks. Content will be iteratively refined through structured feedback from instructors and peers. A detailed outline of session topics, activities, and assignments is provided in Table 1.

The intervention will be delivered through a series of structured training sessions led by bilingual instructors. Sessions will be conducted in a hybrid format, with options for in-person or virtual participation, and will be supplemented by independent work focused on content development and social media engagement. Across the program, each cohort will complete approximately 18 hours of structured training.

To support adherence and minimize attrition, the study will implement a multi-pronged retention strategy that accounts for potential barriers such as scheduling constraints, competing work responsibilities, and other personal obligations. This approach will include flexible scheduling to accommodate participants’ availability, regular reminder communications regarding study activities, and financial compensation for participation.

### Outcomes

Outcomes will be assessed using a combination of surveys, focus groups, social media analytics, and publicly available epidemiologic data to characterize social media practices, evaluate engagement with the training program, and assess the effectiveness of communication strategies.

### Surveys

Surveys will be administered to promotores and members of their social media networks at multiple time points aligned with the stepped-wedge rollout.

At baseline, promotores will complete a 30-minute survey assessing demographics, social media use patterns, and confidence in applying science communication principles related to respiratory virus prevention [28]. The survey will capture social media platform use, including applications used, number of network members, and frequency of reading, posting, and sharing content, as well as experience addressing vaccine-related health claims. These data will be used to describe variability in social media practices, identify emerging platforms for inclusion in analyses, and characterize posting behaviors across community areas. Survey items will draw from the PhenX Toolkit [29], including modules on social determinants of health, COVID-19 exposure, technology access, and media use.

Promotores will also complete brief surveys immediately before and after the training to assess learning related to evaluating online health information, assessing source credibility, and developing evidence-based messages. Post-training surveys will also capture perceived usefulness, acceptability, and feasibility of the training. Implementation outputs will be documented by tracking completion of curriculum assignments and the number and characteristics of infographics and posts produced (e.g., inclusion of credible sources, use of narrative elements).

Additional follow-up surveys will be administered at study conclusion to assess perceived reach, communication experiences, and confidence in applying science communication strategies.

Members of promotores’ social media networks will complete surveys at baseline, at 6-months, and post-campaign to assess exposure to social media content, recall, perceived credibility, and vaccination-related decision-making.

Individuals who wish to receive compensation for completing the surveys will be asked to provide their email address. This contact information will not be stored with survey response data.

Survey data will be collected and managed using REDCap (Research Electronic Data Capture) electronic data capture tools hosted at the University of Iowa [30, 31]. REDCap is a secure, web-based software platform designed to support data capture for research studies, providing 1) an intuitive interface for validated data capture; 2) audit trails for tracking data manipulation and export procedures; 3) automated export procedures for seamless data downloads to common statistical packages; and 4) procedures for data integration and interoperability with external sources.

### Focus Groups

Qualitative data will be collected through focus groups or individual interviews with promotores, depending on participant preference, conducted at baseline and following completion of the intervention. Baseline focus groups and interviews will be 60–90 minutes in duration and will be guided by an interview protocol informed by the Appreciative Inquiry framework, an asset-based approach designed to elicit strengths, opportunities, and communication strategies within local contexts.[32] Discussions will assess health information sources, commonly encountered vaccine-related claims, trust in information sources, perceptions of vaccine safety, and vaccination-related decision-making.

Post-intervention focus groups and interviews will explore experiences with the training program, community responses to social media content, engagement patterns, and examples of successful message dissemination or constructive disagreement.

We expect to have 5 to 10 participants per focus group session. Focus groups will be conducted in English and Spanish and will take place in person or virtually (via Zoom), based on participant preference.

### Social Media Engagement

Social media data will be collected from platforms commonly used within participants’ networks, including Facebook and Instagram. Promotores will be trained to export data from their posts (e.g., Facebook JSON exports) and share these files with the research team. For platforms that do not support direct data export, alternative approaches will be used, including participant-reported metrics or researcher access to social media groups or communication channels, as appropriate.

Engagement metrics will include impressions (i.e., number of users who view a post) and indicators of interaction such as likes, comments, and shares. In addition, the content of promotores’ posts and associated audience responses will be analyzed to characterize features of communication, including sentiment, use of personal narratives, and incorporation of scientific evidence. These measures will be used to examine how characteristics of social media content relate to engagement within promotores’ networks.

### Area-Level Health Data

Publicly available data on vaccination coverage and healthcare utilization will be obtained from the Chicago Data Portal and the Illinois Department of Public Health [33, 34]. These data will be used to assess changes in vaccination coverage over time as clusters transition from control to intervention conditions. Exploratory analyses will also examine area-level influenza-like illness, hospitalizations, and emergency department utilization during the campaign period.

### Sample Size

The primary outcome will be vaccination-related decision-making among members of promotores’ social media networks, including willingness to receive recommended immunizations or recommend them to others.

Sample size estimates were informed by prior studies reporting vaccination coverage ranging from 33.1% to 45.4% in urban populations served by Spanish-speaking community health initiatives [33, 35]. Using these estimates, we calculated the required sample size for a paired design with a two-sided α of 0.05, 80% power, a relative risk of 1.5, and an assumed within-participant correlation coefficient of 0.25. We then applied the approach described by Hemming and Taljaard to account for the stepped-wedge cluster design, incorporating four steps, variable cluster sizes, and an intracluster correlation coefficient of 0.01 [36]. Design effect adjustments were applied to estimate overall and per-cluster sample size requirements.

Based on these calculations, we will recruit a minimum of 35 promotores across seven neighborhoods, organized into four clusters. Under the planned four-step rollout, clusters will sequentially transition from control to intervention conditions at six-month intervals. This sample size will provide 80% power to detect a meaningful difference in vaccination-related decision-making over time, corresponding to an increase from 45% at baseline to 68% during the intervention phase. To achieve adequate power, each promotor will recruit approximately 45 members of their social media network to complete baseline and follow-up surveys.

### Data Analysis

Analyses will be conducted to characterize social media practices, evaluate engagement with the training program, and assess the effectiveness of communication strategies over time. All analyses will account for the stepped-wedge cluster design and repeated measures within participants and clusters.

### Surveys

Descriptive data will summarize participant characteristics, social media use patterns, and baseline measures using appropriate summary statistics. Categorical variables will be reported as counts and percentages, and sparse categories will be combined where appropriate. Continuous variables will be assessed for distributional assumptions and summarized as means with standard deviations or medians with interquartile ranges.

Changes in survey-based outcomes, including knowledge, confidence in applying science communication principles, and vaccination-related decision-making, will be evaluated using generalized linear mixed models (GLMMs). These models will account for clustering at the promotor and neighborhood levels and will include fixed effects for time and intervention status. Relevant demographic and contextual covariates (e.g., age, primary language, and zip code) will be included as appropriate.

Repeated measures analyses will be used to assess changes over time across baseline, 6-months, and post-campaign assessments. Binary outcomes will be analyzed using logistic mixed-effects models, while continuous and ordinal outcomes will be analyzed using linear or cumulative link mixed models, depending on distributional assumptions.

### Focus Groups

Qualitative data from focus groups will be analyzed using the constant comparative method [37]. Transcripts will be coded iteratively, with an initial subset independently coded by multiple researchers to develop a preliminary codebook, followed by independent coding of the remaining transcripts. Discrepancies will be resolved through consensus, and themes will be refined using representative quotations. Data collection and analysis will continue until thematic saturation is achieved; if saturation is not reached, additional interviews may be conducted. Findings will be reported in accordance with the Standards for Reporting Qualitative Research [38].

### Social Media Engagement

Social media engagement will be quantified using platform-derived metrics, including impressions, likes, comments, and shares. Count-based outcomes will be analyzed using Poisson or negative binomial regression models, as appropriate based on dispersion characteristics. Continuous or ordinal survey outcomes will be analyzed using linear or cumulative link mixed models, depending on distributional assumptions. Repeated measures of generalized linear mixed models will be used to assess changes in engagement over time while accounting for clustering within promotores and neighborhood clusters under the stepped-wedge design.

Characteristics of social media posts and audience responses will be extracted using large-language models and multimodal analytic approaches applied to text, images, and video content. These characteristics will include sentiment, discussion of personal experience, presence of scientific evidence, and patterns of agreement or disagreement. These features will be incorporated as independent variables in regression and machine learning–based predictive models to examine their association with engagement outcomes and to identify characteristics of posts associated with higher levels of interaction within promotores’ social networks.

In addition to quantifying the volume of engagement, analyses will examine the nature of engagement by evaluating the content of audience responses to promotores’ posts. These analyses will provide insight into how different types of communication strategies influence both the extent and quality of interaction.

Implementation fidelity will be assessed by quantifying the number and characteristics of posts produced, including adherence to training principles such as inclusion of credible sources and use of narrative elements.

### Area-Level Health Data

Community-level vaccination coverage and healthcare utilization outcomes will be analyzed longitudinally as clusters transition from control to intervention conditions. Mixed-effects models will adjust for time effects and clustering to estimate intervention-associated differences while accounting for the stepped-wedge rollout. Zip code–level indicators will be incorporated to assess potential geographic crossover between clusters.

Exploratory analyses will examine area-level influenza-like illness, hospitalizations, and emergency department utilization during the intervention period, recognizing limited statistical power for these outcomes.

### Exploratory Analyses

Exploratory subgroup analyses may be conducted to assess whether intervention effects vary by participant characteristics, including age, primary language, and baseline social media use. These analyses will be considered hypothesis-generating and interpreted cautiously.

### Interim Analyses

Interim analyses will be conducted at prespecified time points to summarize implementation metrics, engagement patterns, and participant feedback. These analyses will be used to monitor feasibility and fidelity of the intervention. Interim findings will inform operational adjustments (e.g., scheduling or delivery format) but will not be used to modify primary outcomes or determine early trial termination.

### Missing Data

Missing data may occur due to non-adherence or participant attrition over the course of the study. Multiple imputation by chained equations will be used to address missing survey data under the assumption that data are missing at random. Imputation models will incorporate demographic variables, baseline measures, cluster indicators, and time effects. Sensitivity analyses will be conducted to evaluate the robustness of findings to different assumptions about missingness. Results from imputed datasets will be compared with complete case analyses to assess the robustness of findings.

### Blinding

Research personnel responsible for survey administration and epidemiologic data analyses will be blinded to the timing of cluster transitions from control to intervention conditions. Because allocation is non-randomized and occurs at the cluster level, and only outcome assessors and analysts are blinded, formal unblinding procedures will not be required.

### Ethics Approval

This study has been reviewed and approved by the Institutional Review Board at the University of Chicago IRB24–1997, with reliance agreements established as needed for collaborating institutions. All study procedures will be conducted in accordance with applicable ethical guidelines and regulations.

### Risks and Protections

Risks associated with participation are expected to be minimal and may include potential discomfort discussing health-related topics or concerns related to sharing information on social media. Participants will be informed that they may skip questions or decline to participate in any study activity. The study team will take steps to minimize risks by emphasizing voluntary participation and protecting confidentiality.

### Oversight and Monitoring

Oversight of study conduct and participant safety will be guided by an Institutional Review Board–approved Data and Safety Monitoring Plan. Given the minimal-risk, educational nature of the intervention, a formal Data and Safety Monitoring Board will not be convened. Study oversight will be conducted by the Principal Investigator and research team in accordance with institutional and federal requirements.

If an adverse event occurs, the PI will report it promptly to the IRB, institutional compliance offices, and NIH in accordance with federal and institutional requirements. The study team will assess the event to determine its cause and evaluate whether it is related to study activities. When appropriate, the informed consent document and/or study protocol will be revised to incorporate additional safeguards identified through review of the event.

### Confidentiality and Data Security

All study data will be handled in accordance with applicable federal, state, and institutional regulations to ensure participant confidentiality. Prior to participation, promotores will receive an overview of study data collection procedures, including the use of REDCap-based questionnaires, social media engagement metrics, and publicly available epidemiologic data sources.

Each participant will be assigned a unique study identifier. Any files linking personal identifiers to study data will be accessible only to authorized research staff and will be stored separately from analytic datasets. These linking files will be destroyed following completion of data collection and prior to analysis. Identifiers and contact information will not be shared outside the research team.

Survey and social media analytics data will be collected and stored using secure, password-protected systems, including REDCap hosted at the University of Iowa. Qualitative data, including focus group recordings, transcripts, and coding files, will be stored on secure, password-protected institutional systems. Audio, video, and written materials will be assigned unique identifiers and de-identified prior to analysis. Recordings and transcripts will be encrypted, and access will be restricted to authorized study personnel.

Social media data shared by promotores will be limited to content and engagement metrics relevant to study objectives. Any data obtained from social media platforms will be handled in a manner that protects the privacy of both promotores and members of their networks.

De-identified vaccination coverage and healthcare utilization data will be obtained from the Chicago Data Portal and the Illinois Department of Public Health under applicable data use agreements.[34, 39] These data will be stored securely and linked to study datasets using geographic identifiers when permitted. Limited datasets (e.g., dates and zip codes) may be retained for future secondary analyses in accordance with institutional policies.

All publications and presentations will report only de-identified, aggregate data, and no identifying information will be included.

### Dissemination

Findings from this study will be disseminated through peer-reviewed publications and conference presentations. In addition, results will be shared with participating community-based organizations and promotores in accessible formats, including summaries and presentations tailored for community audiences. These dissemination efforts are intended to support ongoing health communication efforts and inform future interventions within Spanish-speaking communities.

In accordance with institutional policies and data use agreements, participant-level data will not be shared publicly to protect confidentiality. Statistical code may be made available upon reasonable request and subject to institutional review.

### Trial Status

At the time of manuscript submission, recruitment has begun and is ongoing, but data collection has not yet been completed. This study is registered on ClinicalTrials.gov (NCT06417762). This manuscript reflects protocol version 3.

## Discussion

This study will evaluate a community-engaged social media intervention designed to support promotores de salud in disseminating evidence-based health information within Spanish-language networks. Building on prior research demonstrating the influence of message characteristics and trusted messengers on health communication, this study aims to address gaps in understanding how health information circulates within these networks and which communication strategies are most effective in promoting engagement and informed decision-making. By integrating training in science communication with real-world application in social media environments, this study will generate evidence on both communication processes and intervention effectiveness.

Several features of the study design strengthen its ability to evaluate communication strategies within real-world settings. The stepped-wedge design allows for evaluation of intervention effects while ensuring that all participating communities receive the intervention over time. The use of promotores further leverages trusted community-based relationships to support message dissemination. The integration of multiple data sources, including surveys, qualitative data, social media analytics, and area-level health data, enables a more comprehensive understanding of both communication dynamics and downstream outcomes than would be possible using a single method alone. Evaluating communication strategies within real-world social media environments also enhances the relevance and potential scalability of the findings.

At the same time, several challenges should be considered in interpreting the results. The non-randomized design may introduce potential confounders, although analytic approaches will account for clustering and time effects. Reliance on self-reported data may introduce reporting bias, and variation in social media platform use and engagement across participants may result in heterogeneous exposure to intervention content. In addition, the study is conducted within a single metropolitan area, which may limit generalizability to other settings. Measurement of community-level outcomes may also be constrained by the availability and granularity of publicly available data.

Despite these considerations, findings from this study will provide important insights into how health information is shared, interpreted, and acted upon within Spanish-language social media networks. By identifying communication approaches that are both acceptable to community health workers and effective in engaging their networks, this work may inform the design of future interventions aimed at improving access to reliable health information and supporting informed decision-making in diverse communities. The integration of community-based expertise with digital communication strategies offers a potentially scalable model for addressing similar challenges in other settings.

## Figures and Tables

**Table 1:** Dime La VerDAD curriculum structure.

Session	Topic	Details	Homework
Focus Group	Baseline Discussion	Structured discussion to understand current social media practices, commonly encountered health claims, and communication experiences	
1	Designing Infographics	Introduction to infographics and visual communication principles; overview of Canva tools	• Join the study communication group (on WhatsApp) • Friend/follow *Dime La VerDAD* on social media platforms • Access the Canva folder • Share 2–3 practice posts on social media
2	Understanding Inaccurate Health Claims	Overview of different types of inaccurate health information and how it circulates online; discussion of potential impacts	• Invite approximately 45 social media network members to complete the pre-survey • Identify one vaccine-related health claim to address ○ Locate 2–3 credible sources • Begin drafting an infographic in Canva
3	Communicating Across Audiences	Principles of effective communication and audience engagement	• Develop draft infographics (1 Facebook, 1 Instagram) ○ Include a link to a credible resource • Draft a caption incorporating a personal narrative ○ Include (2–3) relevant hashtags and tags as appropriate
4	Evaluating Source Credibility	Methods for assessing the reliability of online sources (e.g., SIFT method, fact-checking tools)	• Verify sources using structured evaluation methods ○ Identify additional credible resources if needed • Revise draft infographic and caption based on instructor and cohort participant feedback
5	Applying Science Communication Strategies	Strategies for making evidence-based messages engaging and understandable	• Refine your infographic and caption based on feedback • Post standardized informational content provided by the study team
6	Responding to Disagreement on Social Media	Approaches to addressing dissent constructively and maintaining professionalism online	• Develop a strategy to address responses from people with opposing views • Present final infographic and communication strategy • Post standardized informational content provided by the study team • Post your finalized infographic and caption • Six months after baseline focus group, invite social media network members to complete the post-survey

## Data Availability

Data supporting the findings of this study will be made available upon reasonable request to the corresponding author, in accordance with the National Institutes of Health Data Management and Sharing Policy. De-identified individual participant data and a data dictionary will be shared following publication, contingent upon approval of a data use agreement and in compliance with institutional review board and privacy regulations.
